# ERK1/2 and p38 MAPKs Are Complementarily Involved in Estradiol 17ß-d-Glucuronide-Induced Cholestasis: Crosstalk with cPKC and PI3K

**DOI:** 10.1371/journal.pone.0049255

**Published:** 2012-11-14

**Authors:** Andrea C. Boaglio, Andrés E. Zucchetti, Flavia D. Toledo, Ismael R. Barosso, Enrique J. Sánchez Pozzi, Fernando A. Crocenzi, Marcelo G. Roma

**Affiliations:** Institute of Experimental Physiology, National Scientific and Technical Research Council/National University of Rosario, Rosario, Argentina; University of Valencia, Spain

## Abstract

**Objective:**

The endogenous, cholestatic metabolite estradiol 17ß-d-glucuronide (E_2_17G) induces endocytic internalization of the canalicular transporters relevant to bile formation, Bsep and Mrp2. We evaluated here whether MAPKs are involved in this effect.

**Design:**

ERK1/2, JNK1/2, and p38 MAPK activation was assessed by the increase in their phosphorylation status. Hepatocanalicular function was evaluated in isolated rat hepatocyte couplets (IRHCs) by quantifying the apical secretion of fluorescent Bsep and Mrp2 substrates, and in isolated, perfused rat livers (IPRLs), using taurocholate and 2,4-dinitrophenyl-*S*-glutathione, respectively. Protein kinase participation in E_2_17G-induced secretory failure was assessed by co-administering selective inhibitors. Internalization of Bsep/Mrp2 was assessed by confocal microscopy and image analysis.

**Results:**

E_2_17G activated all kinds of MAPKs. The PI3K inhibitor wortmannin prevented ERK1/2 activation, whereas the cPKC inhibitor Gö6976 prevented p38 activation, suggesting that ERK1/2 and p38 are downstream of PI3K and cPKC, respectively. The p38 inhibitor SB203580 and the ERK1/2 inhibitor PD98059, but not the JNK1/2 inhibitor SP600125, partially prevented E_2_17G-induced changes in transporter activity and localization in IRHCs. p38 and ERK1/2 co-inhibition resulted in additive protection, suggesting complementary involvement of these MAPKs. In IPRLs, E_2_17G induced endocytosis of canalicular transporters and a rapid and sustained decrease in bile flow and biliary excretion of Bsep/Mrp2 substrates. p38 inhibition prevented this initial decay, and the internalization of Bsep/Mrp2. Contrarily, ERK1/2 inhibition accelerated the recovery of biliary secretion and the canalicular reinsertion of Bsep/Mrp2.

**Conclusions:**

cPKC/p38 MAPK and PI3K/ERK1/2 signalling pathways participate complementarily in E_2_17G-induced cholestasis, through internalization and sustained intracellular retention of canalicular transporters, respectively.

## Introduction

Bile formation is a highly regulated process that depends upon the coordinated transport activity of basolateral and canalicular carriers on the hepatocyte. Complex signalling pathways regulate the density and activity of these carriers, and its imbalance impairs secretory function, leading to retention of biliary components both in liver and blood [1].

Estradiol 17ß-D-glucuronide (E_2_17G) is an endogenous metabolite of 17ß-estradiol that induces an acute, reversible cholestasis in rats [2]. Since its level build up during pregnancy, it has been suggested to be relevant to the pathogenesis of intrahepatic cholestasis in pregnant, susceptible women [2].

The mechanisms involved in E_2_17G induces cholestasis seems to be multifactorial. Trans-inhibition by E_2_17G of Bsep-mediated canalicular transport of bile salts [3] and increase in paracellular permeability leading to dissipation of plasma-to-bile osmotic gradients [4] have been shown to be causal factors. In addition, our group showed that endocytic internalization of both Bsep [5] and Mrp2 [6], [7], two canalicular transporters crucial for bile formation, is also a key cholestatic mechanism. In addition, our group also demonstrated that E_2_17G activates both classical (Ca^2+^-dependent) protein kinase C (cPKC) isoforms [8] and the phosphoinositide 3-kinase (PI3K)-Akt signalling pathway [9], and that these signalling events are cooperatively involved in the changes in Bsep/Mrp2 localization status. Whereas cPKC triggers endocytic internalization of these transporters, PI3K-Akt retains them into intracellular, vesicular compartments [8], [9].

Since the biological effects of cPKC and PI3K are often mediated through downstream protein kinases, we speculated that other signalling mediators are involved, provided they can crosstalk with cPKC or PI3K. Likely candidates are the mitogen activated protein kinases (MAPKs). Both cPKC [10], [11] and PI3K/Akt [12], [13] often act as upstream signalling activators of MAPKs in hepatocytes.

The main vertebrate MAPKs are the extracellular signal-regulated kinases 1 and 2 (ERK1/2), c-Jun amino-terminal kinases 1 and 2 (JNK1/2), and p38 kinase (isoforms α and β in hepatocytes). ERK1/2 is preferentially activated by growth factors, while JNK1/2 and p38 are more responsive to stress stimuli [14]. After recognition of these effectors by surface receptors, MAPKs are activated by three-tiered, sequential phosphorylations mediated by small GTP-binding proteins (*e.g.*, Ras, Rap) and two protein kinases (MAPKKK and MAPKK), which act as dual-specificity enzymes that activate a selective MAPK type. Non-canonical activation of MAPKs involves MAPK autophosphorylation, or direct MAPK phosphorylation by alternative protein kinases, such as Src or ZAP70 [15].

Supporting MAPK involvement in E_2_17G-induced cholestasis, 17ß-estradiol, a E_2_17G precursor which evokes in hepatocyte-derived cell lines most signalling pathways activated by E_2_17G (including the cholestatic ones, cPKC [16] and PI3K [17]), activates MAPKs in several cell types, such as cardiomiocytes [18], neurons [19], and cholangiocytes [20]. Furthermore, many rapid responses of estrogens are mediated by MAPKs, particularly of the ERK1/2 and p38 MAPK types [21]. Even more suggestively, the cholestatic bile salt taurolithocholate (TLC), which shares with E_2_17G the capability to evoke the pro-cholestatic route mediated by PI3K [22], activates p38 in isolated rat hepatocytes [23].

These findings have prompted us to study the role for MAPKs in E_2_17G-induced cholestasis. In particular, we ascertained which MAPK types contribute to E_2_17G-induced cholestasis both in isolated rat hepatocyte couplets (IRHCs) and in the isolated, perfused rat liver (IPRL). Our results demonstrate that both p38 and ERK1/2 contribute to the impairment of localization and function of Bsep and Mrp2 induced by E_2_17G, by acting in a complementary manner, and downstream of cPKC and PI3K, respectively.

## Materials and Methods

### Materials

Cholyl-glycylamido-fluorescein (CGamF) was a generous gift from Prof. Alan Hofmann (University of California, San Diego). E_2_17G, collagenase type A (from Clostridium histolyticum), bovine serum albumin (BSA), trypan blue, L-15 culture medium, dimethyl sulfoxide (DMSO), Triton X-100, ethylene glycol tetraacetic acid, sodium dodecyl sulfate, tetramethylethylenediamine, dithiothreitol, ammonium persulfate, urethane, protease inhibitor cocktail, β nicotinamide adenine dinucleotide hydrate and, 1-chloro-2,4-dinitrobenzene (CDNB) were from Sigma Chemical Co. (St. Louis, MO, USA). 5-Chloromethylfluorescein diacetate (CMFDA) was from Molecular Probes (Eugene, OR, USA). Gö6976, SB203580, PD980589, SP 600125, and sodium taurocholate were from Calbiochem (San Diego, CA, USA). Wortmannin (WM) was from Fluka AG (Buchs, Switzerland). Cell lysis buffer was from Cell Signaling Technology (Beverly, MA). The chemiluminescence reagent, and Hyperfilm ECL were from Thermo Fisher Scientific, Inc. (Waltham, MA, USA). All the other reagents were of analytical-grade.

### Ethics Statement

All animals received humane care according to the criteria outlined in the “Guide for the Care and Use of Laboratory Animals” Eight Edition (National Academy of Sciences, 2011). Experimental procedures were carried out according to the local Guideline for the Use of Laboratory Animals (Resolution N° 6109/012) established by the institutional Bioethical Committee for the Management of Laboratory Animals and approved by the Faculty of Biochemical and Pharmaceutical Sciences of the National University of Rosario. A system of local committee-based regulatory control offers an equivalent level of regulatory control to that exercised by the systems in Canada. The guidelines for the use of laboratory animals has been approved by our Faculty in 2002, based on CCAC (Canadian Council on Animal Care) guidelines documents. The document has been updated since then according to current and emerging issues for the research community and to advances in laboratory animal care.

### Animals

Adult female Wistar rats weighing 250–300 g and bred in our animal house as described [24], were used in all studies. Treatment were carried out under urethane anesthesia (1 g/kg intraperitoneally), and maintained thus throughout. When necessary, body temperature was measured with a rectal probe and maintained at 37°C.

### Isolation and Culture of IRHCs

To obtain a preparation enriched in IRHCs, livers were perfused according to the two-step collagenase perfusion procedure, and further enriched by centrifugal elutriation [25]. Cell viability, assessed by trypan blue exclusion, was greater than 90%. To allow for restoration of their polarity, IRHCs were plated onto 24-well plastic plates at a density of 0.5×10^5^ U/mL in L-15 culture medium, and cultured for 5 h.

### IRHC treatments

IRHCs were exposed to the vehicle (DMSO; control group) or E_2_17G (200 µM) for 20 min. To evaluate the role of MAPKs in E_2_17G-induced hepatocanalicular dysfunction, IRHCs were preincubated with SB203580 (1 µM), a p38α/ß inhibitor, PD980589 (5 µM), an inhibitor of the MAPKK upstream of ERK1/2 (MEK1/2), or SP600125 (1 µM), a JNK1/2 inhibitor, for 15 min., followed by addition of E_2_17G for another 20-min period. Co-inhibition of cPKC and PI3K was carried out by co-administering their specific inhibitors Gö6976 (1 µM) and WM (100 nM), respectively.

### Function and localization of Bsep and Mrp2 in IRHCs

Functional changes in Bsep and Mrp2 were evaluated by assessing the canalicular vacuolar accumulation (cVA) of CGamF, a fluorescent Bsep substrate [26], [27], or glutathione methylfluorescein (GS-MF), a fluorescent Mrp2 substrate derived from CMFDA; this latter compound diffuses passively, and is intracellularly metabolized by esterases and glutathione S-transferases to render GS-MF [28].

For transport studies, cells were washed twice with L-15 and exposed to 0.3 µM CGamF [26] or 2.5 µM CMFDA [29] for 15 min; after that, images were captured using a digital camera (Q-color5; Olympus America, Center Valley, PA) under a fluorescence microscopy (Zeiss Axiovert 25). cVA was assessed by determining the proportion of IRHCs (>200 per preparation) accumulating in their canalicular vacuoles the fluorescent compounds, as described [29]. Intracellular localization of Bsep and Mrp2 was evaluated by confocal laser microscopy (Zeiss Pascal LSM 5, Carl Zeiss, Walldorf, Germany). For this purpose, cells were fixed with 4% paraformaldehyde in phosphate-buffered saline, and immunostained with a rabbit anti-rat Bsep (1∶100, 2 h; Kamiya Biomedical Co., Seattle, WA, USA), or with a mouse anti-human MRP2 (1∶100, 2 h; [M2III-6], Alexis Biochemicals, San Diego, CA, USA), followed by incubation with a Cy2-conjugated donkey anti-rabbit IgG or a Cy2-conjugated donkey anti-mouse IgG (1∶200, 40 min; Jackson ImmunoResearch Laboratory, West Grove, PA). Densitometric analysis of confocal microscopy images was performed along a line perpendicular to the canalicular vacuole with ImageJ 1.34 m, as described [8]. The canalicular space on Bsep/Mrp2-labeled IRHCs was identified by superposing each fluorescent image with its respective differential interface contrast (DIC) image [5].

### Colocalization of Mrp2/Bsep with Rab11a in IRHCs

Colocalization studies of Mrp2 and Bsep with Rab11a were performed as previously described [30]. Transporters were stained using the primary and secondary antibodies described above, and Rab11a was detected by using two different specific antibodies: a mouse monoclonal to Rab11a antibody (1∶100, 2 h; Abcam, Cambridge, MA) for colabeling with Bsep and a rabbit polyclonal to Rab11a antibody (1∶100, 2 h; Invitrogen) for colabeling with Mrp2, followed by incubation with a Cy3-conjugated donkey anti-mouse IgG and a Cy3-conjugated goat anti-rabbit IgG (1∶200 1 h; Jackson ImmunoResearch Laboratory, West Grove, PA), respectively. To delimit the canalicular vacuoles, F-actin was stained by adding Alexa Fluor 635 phalloidin (1∶80, 1 h; Invitrogen, Carlsbad CA) together with the secondary antibodies for Mrp2, Bsep and Rab11a.

### Western blot analysis of MAPK phosphorylation

Activation of p38, ERK1/2, and JNK1/2 was assessed by evaluating by Western blotting the phosphorylation status of these MAPKs in lysates of primary-cultured rat hepatocytes. Briefly, isolated rat hepatocytes were obtained by collagenase perfusion, as described [31], and cultured in 3-cm Petri dishes at a density of 2×10^6^ cells/mL. After 5 h of culture, cells were exposed to E_2_17G (200 µM) for 10–60 min, washed with cold phosphate-buffered saline, and finally resuspended in cell lysis buffer and a protease inhibitor cocktail. Aliquots containing an equivalent total protein content, as determined by the Lowry procedure with BSA as the standard [32], were subjected to sodium dodecyl sulfate/12% polyacrylamide gel electrophoresis, electrotransferred to Immobilon-P membranes and probed overnight with a rabbit anti-phosphorylated p38 (1∶300; D-8, Santa Cruz, CA, USA), a rabbit anti-phosphorylated ERK1/2 (1∶1000; Cell Signaling), and a mouse anti-phosphorylated JNK1/2 (1∶2000; Cell Signaling) antibodies. Stripped membranes were reprobed with a rabbit anti-ERK1/2 antibody (1∶1000; Cell Signaling), a rabbit anti-JNK1/2 antibody (1∶1000; Cell Signaling) or a mouse anti-p38 (1∶500; N-20, Santa Cruz Biotechnology). After using a donkey anti-rabbit IgG secondary antibody or a goat anti-mouse IgG (1∶5000, 1 h; Thermo Fisher Scientific, Waltham, MA, USA), a chemiluminescence reagent, and Hyperfilm ECL, phosphorylated and total MAPKs bands were quantified by densitometry with ImageJ 1.34 m.

### Studies in IPRLs

Livers from bile duct-cannulated rats (Intramedic PE-10 tubing, Clay Adams) were perfused *in situ*, as described elsewhere [8]. Transport activity of Mrp2 and Bsep in IPRL was evaluated by measuring 2,4-dinitrophenyl-*S*-glutathione (DNP-SG) and taurocholate excretion, respectively. For this purpose, sodium taurocholate (2 µM) and CDNB (0.5 µM) were added to the perfusion medium.

After a 20-min equilibration period, the p38 inhibitor SB203580 (250 nM) or the ERK1/2 inhibitor PD98059 (5 µM) or their solvent (DMSO; 370 µL/L) were added to the reservoir. Fifteen min later, a 5-min basal bile sample was collected, followed by administration of E_2_17G (3 µmol/liver; single intraportal injection, over a 1-min period), or its solvent in controls [DMSO/10% BSA in saline (4∶96)]. Bile was then collected at 5-min intervals for another 30-min period. Experiments were considered valid only if the initial bile flow was greater than 30 µL/min/kg of body weight. Liver viability was evaluated by monitoring lactate dehydrogenase release into the perfusate outflow [33]; experiments exhibiting activities over 20 U/L were discarded.

DNP-SG content in bile was measured by high-performance liquid chromatography, as described [34]. Biliary bile salt concentration was determined by a modification of the Talalay's method [35].

At the end of the perfusion period, a liver lobe was excised, frozen in isopentane precooled in liquid nitrogen, and stored at −80°C for further immunofluorescence and confocal microscopy analysis of Mrp2 and Bsep intracellular localization. F-actin staining was carried out to demarcate the limits of the canaliculi, as described [5], [8]. Liver sections were fixed and stained as described [6], [7], followed by overnight incubation with the specific antibodies against Bsep or Mrp2, and 1 h incubation with the appropriate cyanine 2-conjugated secondary antibodies, and Alexa Fluor 568 phalloidin (1∶100, 1 h) for F-actin. To ensure comparable staining and image capture performance for the different groups belonging to the same experimental protocol, liver slices were prepared on the same day, mounted on the same glass slide, and subjected to the staining procedure and confocal microscopy analysis simultaneously. Quantification of the degree of Bsep and Mrp2 endocytic internalization was performed on confocal images using ImageJ 1.34 m (National Institutes of Health), as described [5].

### Statistical Analysis

Results are expressed as mean ± standard error of the mean (SEM). Statistical analysis was performed with one-way analysis of variance followed by the Newman-Keuls test. The variances of the densitometric profiles of Bsep and Mrp2 localization were compared with the Mann-Whitney U test. P values<0.05 were considered to be statistically significant.

## Results

### E_2_17G activates MAPKs

**Figure 1:**
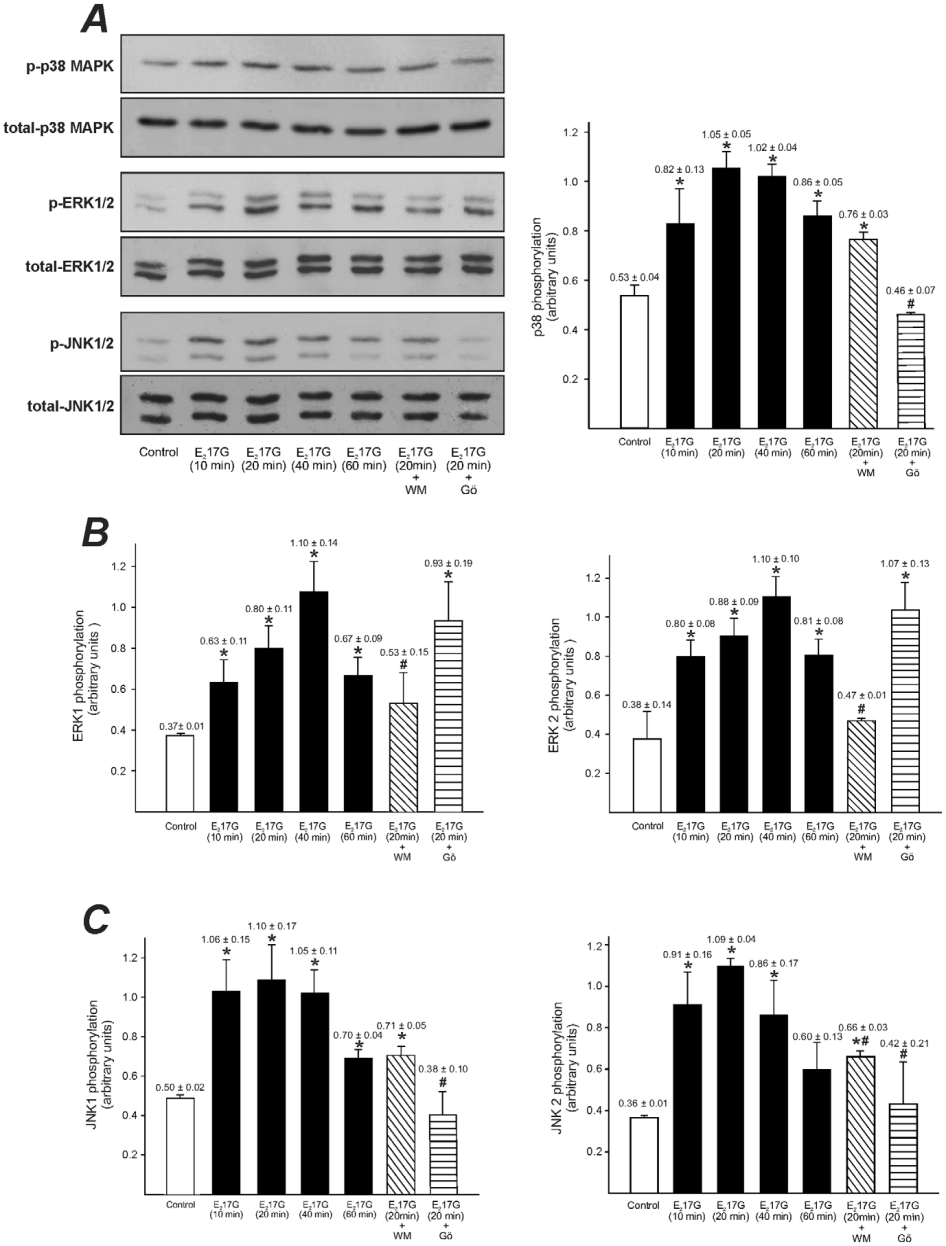
E_2_17G activates the MAPK signalling pathway. *(A)* left panel: representative Western blottings of phospho (p)-p38, p-ERK1/2, p-JNK1/2 and total forms of all these MAPK types were obtained from whole cellular lysates of primary-cultured rat hepatocytes incubated with E_2_17G (200 µM) for 10 to 60 min, or with E_2_17G (200 µM) for 20 min in cells pretreated with the PI3K inhibitor wortmanin (WM, 100 nM) or with the cPKC inhibitor Gö6976 (Gö, 1 µM) for 15 min. *A* (right panel), and *B* and *C panels* show phosphorylation status of all MAPK types evaluated (calculated as the p-MAPK to total MAPK ratio for each experimental condition). An arbitrary value of 100 was assigned to the band of highest densitometric intensity in every Western blot before the ratio was calculated. The results are shown as mean ± SEM (*n* = 5). *P<0.05 *vs.* control (cells treated only with DMSO), and ^#^P<0.05 *vs.* E_2_17G (20 min).

Western blots of phospho(p)-p38 (Fig. 1; *A*, right panel), p-ERK1/2 (Fig. 1; *B*) and p-JNK1/2 (Fig. 1; *C*) showed that E_2_17G increased the amount of each p-MAPK in a time-dependent manner, with increments becoming apparent as soon as 10 min after E_2_17G administration. Pretreatment with the cPKC inhibitor Gö6976 (1 µM) or the PI3K inhibitor WM (100 nM) selectively prevented the increase in p-p38 and p-ERK1/2, respectively (Fig. 1), indicating that activation of p38 depends upon cPKC whereas activation p-ERK1/2 depends upon PI3K. Instead, pretreatment with either WM or Gö6976 prevented the increase in JNK2 phosphorylation induced by E_2_17G; although a clear trend towards prevention of JNK1 phosphorylation was also observed, this prevention only achieved statistical significance for Gö6976.

### E_2_17G-induced impairment of Bsep and Mrp2 transport function and localization in IRHCs involves p38- and ERK1/2-dependent, additive mechanisms

**Figure 2:**
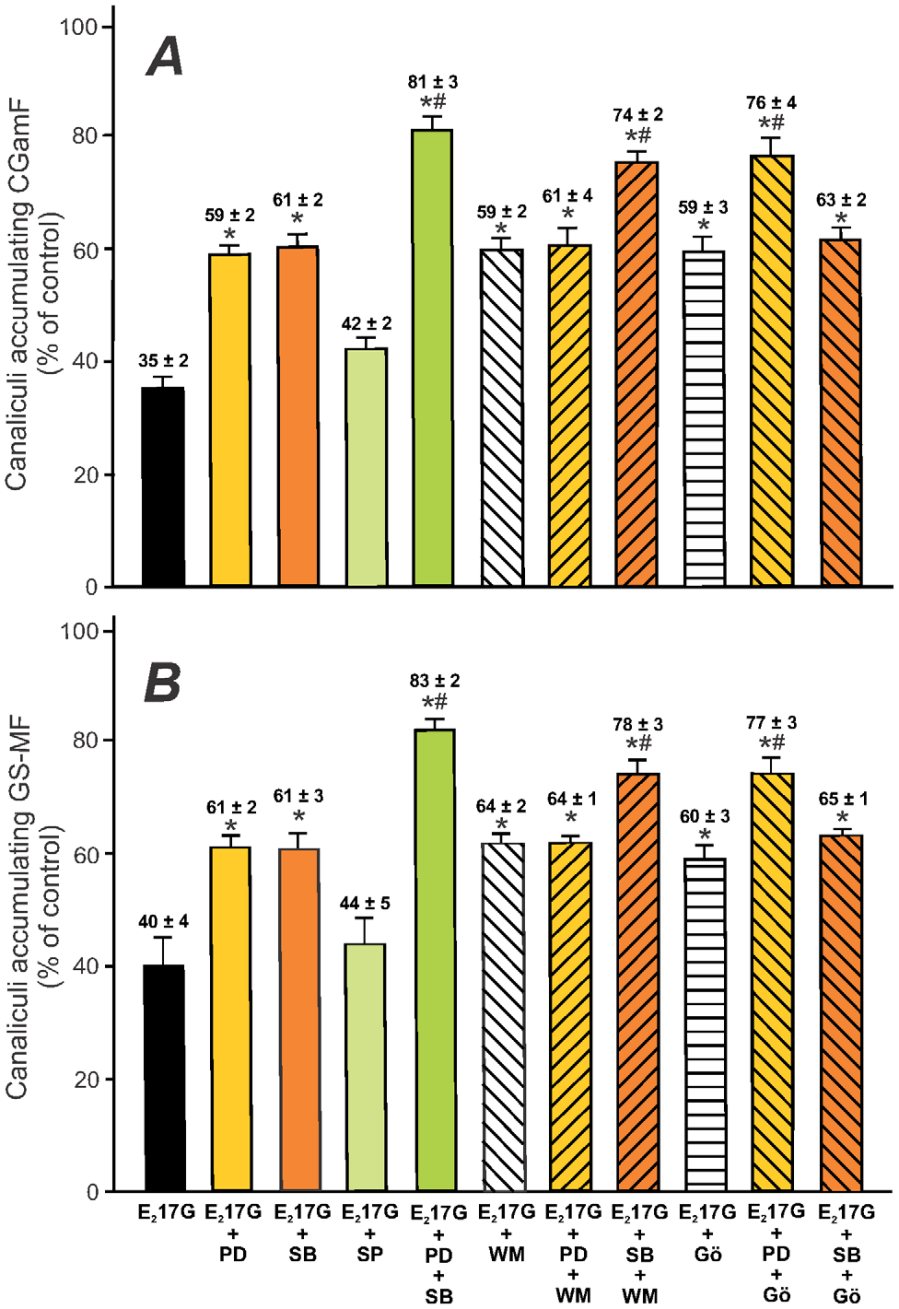
Effect of the inhibition of p38, ERK1/2 and JNK1/2, or the coinhibition of cPKC-ERK1/2, PI3K-p38, or p38-ERK1/2, on E_2_17G-induced impairment of the canalicular accumulation of the Bsep and Mrp2 fluorescent substrates in IRHCs. IRHCs were incubated with E_2_17G (200 µM, 20 min) (or DMSO in controls), with or without pretreatment for 15 min with the JNK1/2 inhibitor SP600125 (1 µM), the ERK1/2 inhibitor PD98059 (PD; 5 µM), and/or the p38 inhibitor SB203580 (SB; 1 µM), together or not with the cPKC inhibitor Gö6976 (Gö; 1 µM) or PI3K inhibitor wortmanin (WM; 100 nM). Canalicular accumulation CGamF (Bsep substrate, panel A) and GS-MF (Mrp2 substrate, panel B) was determined as the percentage of couplets displaying visible fluorescence in their canalicular vacuoles from a total of at least 200 couplets per preparation. The results are expressed as percentages of the control group and are shown as mean ± SEM (*n* = 3–4). *P<0.05 *vs.* E_2_17G, and ^#^P<0.05 *vs.* E_2_17G-WM, E_2_17G-Gö, E_2_17G-PD or E_2_17G-SB.

Functional studies in IRHCs revealed that both the p38 inhibitor SB203580 and the ERK1/2 inhibitor PD98059 significantly prevented E_2_17G-induced impairment in cVA of both the Bsep and the Mrp2 substrates (CGamF and GS-MF, respectively; Fig. 2). Contrarily, the JNK1/2 inhibitor SP600125 was without effect, suggesting that JNK1/2 activation does not play a causal role in E_2_17G-induced cholestasis.

**Figure 3:**
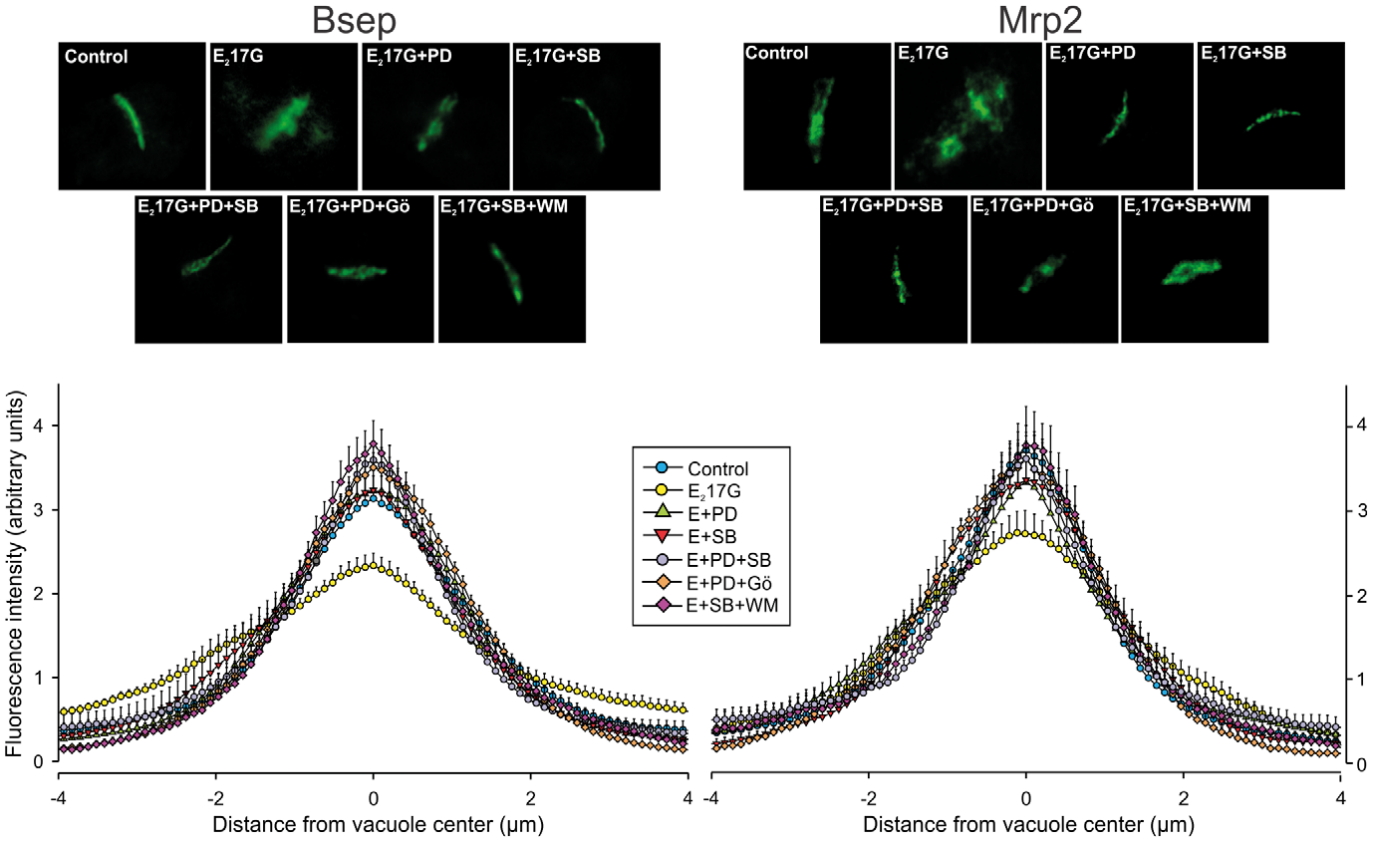
Effect of inhibition of p38 or ERK1/2, and coinhibition of cPKC-ERK1/2, PI3K-p38, or p38-ERK1/2 on E_2_17G-induced retrieval of Bsep and Mrp2 in IRHCs. The upper panels show representative confocal immunofluorescence images of the localization of Bsep and Mrp2 in DMSO-treated (control) or E_2_17G (200 µM)-treated IRHCs, with or without the p38 inhibitor SB203580 (SB; 1 µM) or the ERK1/2 inhibitor PD98059 (PD; 5 µM), in combination or not with the cPKC inhibitor Gö6976 (Gö; 1 µM) or PI3K inhibitor wortmanin (WM; 100 nM). The lower panels show the densitometric analysis of the fluorescence intensity along a line (8 µm) perpendicular to the center of the canalicular vacuole (from +4 to −4 µm). The statistical analysis of the profiles of fluorescence showed a significant change in the E_2_17G-treated group (P<0.05; number of analyzed canalicular vacuoles >10), but this reverted to normal in the E_2_17G-SB, E_2_17G-PD, E_2_17G-PD-SB, E_2_17G-Gö-PD and E_2_17G-WM-SB groups for Bsep and Mrp2.

The effect of E_2_17G on Bsep and Mrp2 transport activity was accompanied by changes in the localization status of these transporters (Fig. 3, top panels). In control IRHCs, the carrier-associated fluorescence was localized mainly in the canalicular vacuoles, whereas in the E_2_17G-treated group, there was extensive relocalization of the fluorescence from the canalicular zone to the cellular body, indicating endocytosis of the canalicular carriers. This phenomenon was markedly prevented by either p38 or ERK1/2 inhibition (Fig. 3, top panels). This was confirmed by densitometric analysis, which showed a flatter Bsep and Mrp2 fluorescence profile in E_2_17G-treated IRHC (Fig. 3, lower panels). ERK1/2 or p38 inhibition prevented partial or totally this relocalization, as densitometric curves were statistically different from that of E_2_17G alone (Fig. 3, lower panels).

The preventive effects of PD98059 and SB203580 on CGamF and GS-MF secretory failures were additive in nature (Fig. 2), suggesting that ERK1/2 and p38 act through different but complementary mechanism. However, additivity of effects can only be assumed when recorded at concentrations of the inhibitors producing maximal effects individually. This was actually the case, since the protective effects of each of them remained virtually the same at concentrations 5-time higher than those used here in additivity studies (data not shown).

### cPKC-p38 and PI3K-ERK1/2signalling pathways are involved in E_2_17G-induced canalicular secretory failure in an additive manner

We have previously demonstrated that the cholestatic effect of E_2_17G is partially prevented by the selective inhibition of cPKC [8] and of PI3K [9], and that these kinases play a partial and complementary role in E_2_17G-induced cholestasis. Since we demonstrated here that cPKC and PI3K are selectively involved in p38 and ERK1/2 activation, respectively (Fig. 1), we tried to reproduce these selective dependencies in the functional field (Fig. 2). As expected, there was a lack of additivity in the protective effects when Gö6976 (cPKC inhibitor) and SB203580 (p38 inhibitor) were added together, and the same holds true when WM (PI3K inhibitor) was added together with PD98059 (ERK1/2 inhibitor). On the other hand, adittivity was observed when cross-combinations of these inhibitors were used (*i.e.*, Gö6976 plus PD98059, or WM plus SB203580). Besides, the pretreatment of IRHCs with these same combinations of inhibitors markedly prevented the E_2_17G-induced internalization of canalicular carriers (Fig. 3, upper panels), resulting in control-like densitometric curves of Bsep/Mrp2 localization (Fig. 3, lower panels). This supports the fact that cPKC-p38 and PI3K-ERK1/2 signalling pathways act in parallel and in a complementary manner to account for E_2_17G cholestatic effects.

### p38-ERK1/2 co-inhibition prevents the colocalization of Bsep/Mrp2 with the endosomal protein Rab11a induced by E_2_17G

**Figure 4:**
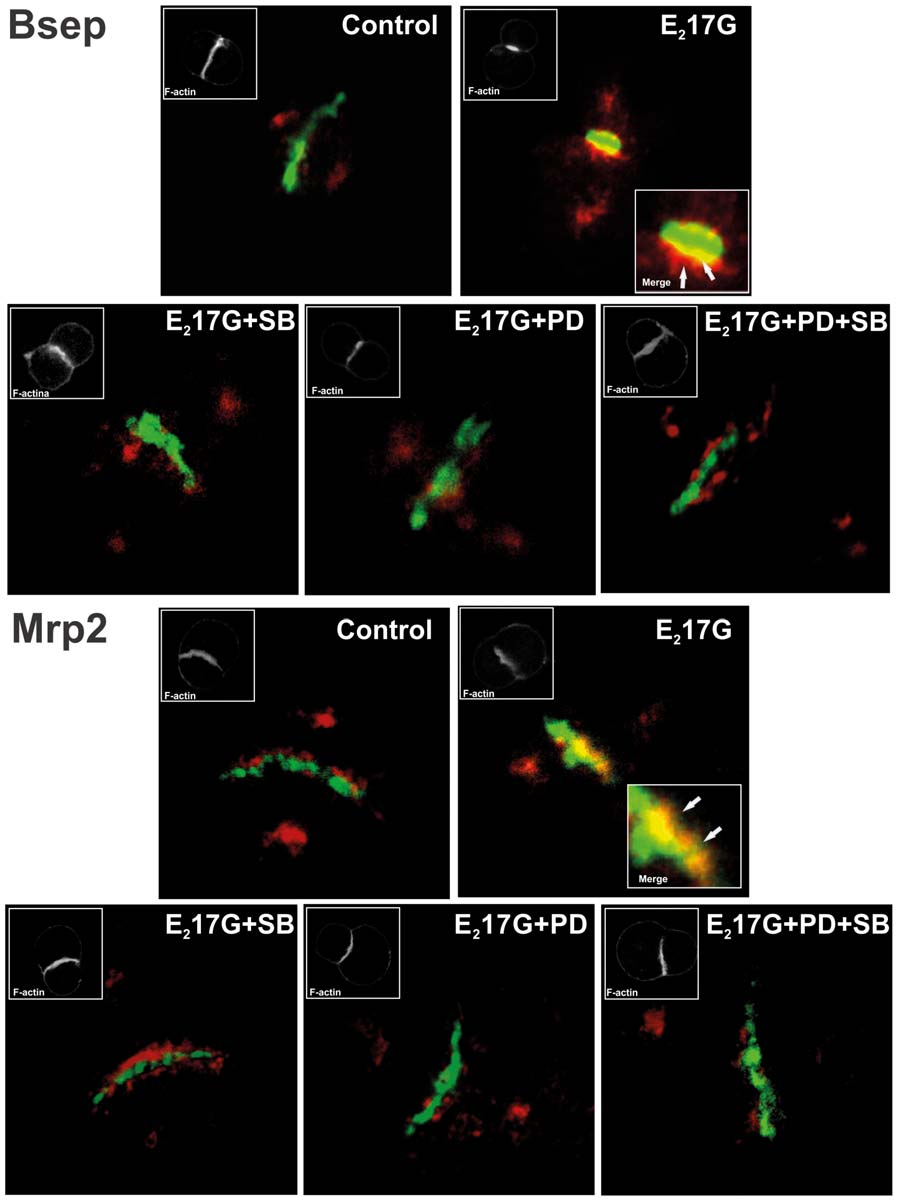
Effect of E_2_17G on colocalization of Rab11a with Mrp2 or Bsep in IRHCs. Immunofluorescence confocal images showing staining of Mrp2 or Bsep (green) and Rab11a (red) in IRHCs treated with DMSO (control), the ERK1/2 inhibitor PD98059 (PD; 5 µM), the p38 inhibitor SB203580 (SB; 1 µM), or both inhibitors together. Colocalization of Rab11a with Mrp2 or Bsep in the E_2_17G-treated group is indicated by orange-yellow fluorescence in merged images. Insets depict F-actin staining, which was used to demarcate the limits of the canalicular vacuoles.

In E_2_17G cholestasis, canalicular carriers are rapidly endocytosed from the canalicular membrane, and can return back to this membrane, presumably from the apical recycling endosomes (ARE) [1]. To visualize this phenomenon, and its prevention by MAPK inhibitors, we evaluated in IRHCs the colocalization status of canalicular carriers with the endosomal protein Rab11a, a marker of ARE that has been shown to colocalize with apically endocytosed proteins in hepatocytes [36]. Using this approach, no colocalization was observed between Rab11a (red) and Bsep or Mrp2 (green) in controls (Fig. 4). Contrarily, E_2_17G induced colocalization of canalicular carriers with Rab11a (yellow/orange merge). IRHCs pretreated with either SB203580 or PD98059 showed lack of colocalization, as in controls; this indicates that either or both the carrier internalization towards ARE was prevented or the carrier reinsertion from ARE has been accelerated.

### p38 is involved in the initial impairment of bile secretory function induced by E_2_17G, whereas ERK1/2 blocks its recovery in the IPRL model

The IRHC model revealed the existence of two complementary mechanisms accounting for E_2_17G-induced cholestasis, which differentially depends upon the cPKC/p38 and PI3K/ERK1/2 pathways (see above). The nature of these two cholestatic mechanisms cannot however be ascertained by using this approach. Using the IPRL model, which unlike IRHCs allow for the dissection of mechanisms occurring separately in time, we had shown a differential role for cPKC and PI3K in E_2_17G-induced cholestasis: whereas cPKC is involved in the initial reduction in bile flow due to transporter endocytosis, PI3K (via Akt) blocks the otherwise spontaneous reinsertion of the endocytosed transporters [9]. We therefore used this model to assess whether p38 and ERK1/2 reproduce the cholestatic mechanisms induced by their respective upstream activators, cPKC and PI3K.

**Figure 5:**
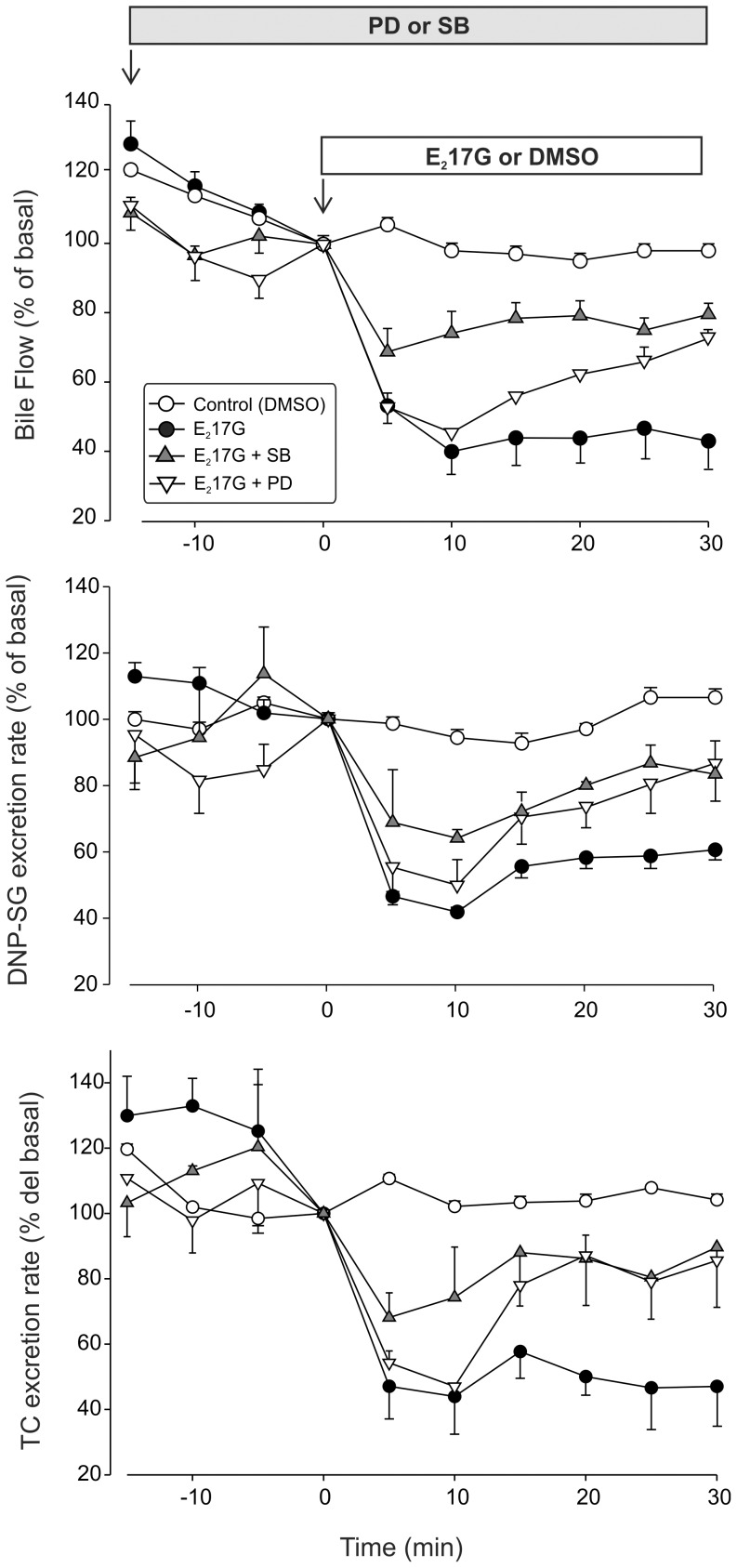
Effect of inhibition of p38 or ERK1/2 on E_2_17G-induced decrease of bile flow and biliary secretion of the Mrp2 and Bsep substrates DNP-SG and taurocholate, respectively, in the perfused rat liver (IPRL) model. IPRLs were treated with a portal bolus of E_2_17G (2 µmol/liver), or with the E_2_17G vehicle DMSO (control), in the presence and absence of the ERK1/2 inhibitor PD98059 (PD; 5 µM) or the p38 inhibitor SB203580 (SB; 250 nM). The effect of the treatments on (*A*) bile flow, (*B*) DNP-SG excretion, (*C*) and taurocholate excretion are shown. Results are expressed as the mean ± SEM (*n* = 4).

The bolus administration of E_2_17G induced a 61% decrease in bile flow within 10 min, which did not recover throughout the perfusion period (Fig. 5, upper panel). This was accompanied by a decrease in the biliary excretion of the Mrp2 and Bsep substrates DNP-SG and taurocholate, respectively (Fig. 5, middle and lower panels). Whereas the p38 inhibitor SB203580 (250 nM) prevented this initial drop, the ERK1/2 inhibitor PD98059 (5 µM) had little, if any, effect on this event. In contrast, PD98059 accelerated the recovery of both bile flow and biliary excretion of Mrp2 and Bsep substrates from 15 minutes of E_2_17G administration onwards. SB203580 and PD98059, when added alone, did not induce any changes in these parameters (data not shown).

**Figure 6:**
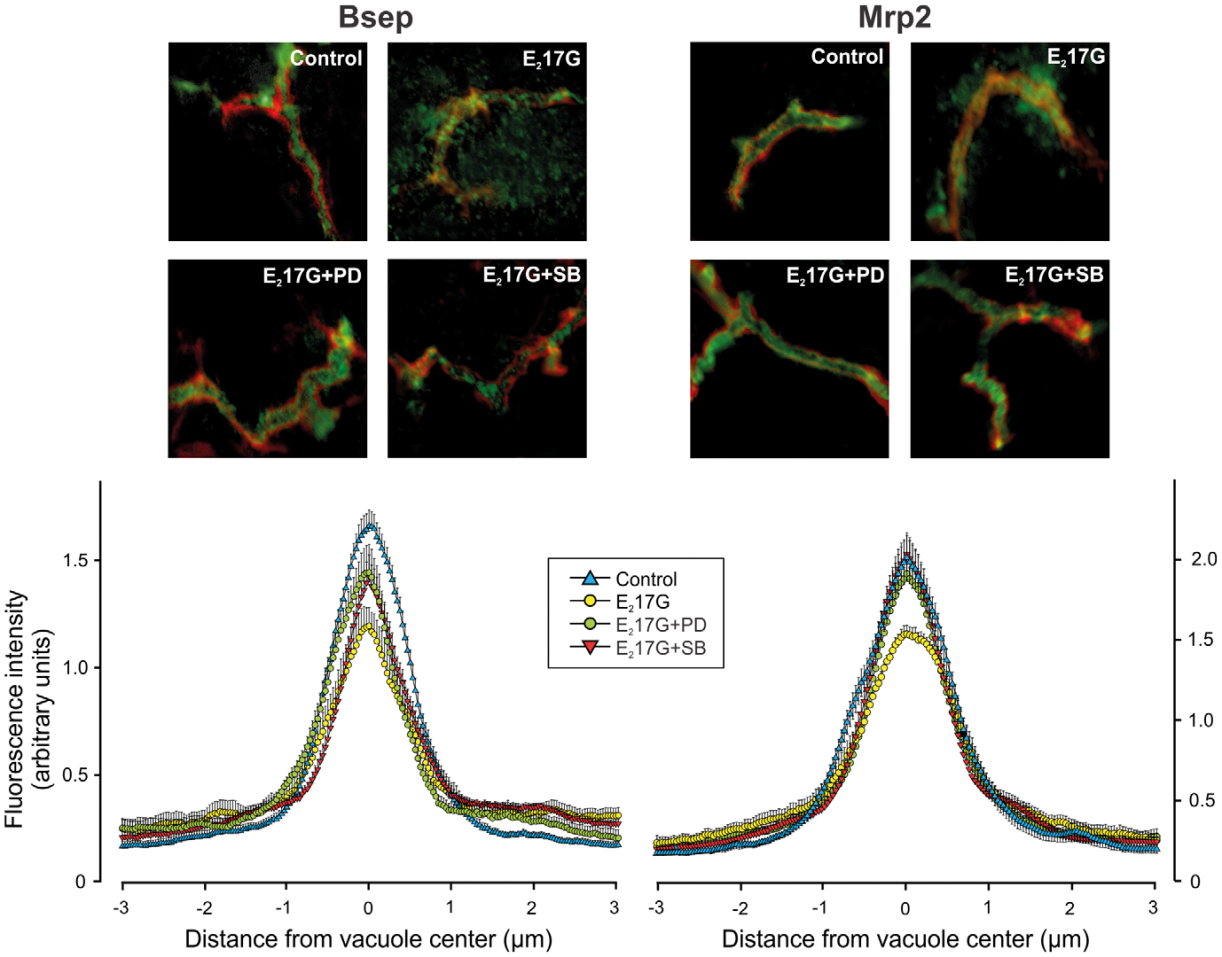
Effect of inhibition of p38 or ERK1/2 on E_2_17G-induced retrieval of Bsep and Mrp2 in perfused rat livers at the end of the perfusion period. The upper panels show representative confocal images showing co-staining of Mrp2 or Bsep (green) with F-actin (red; used to visualize the bile canaliculus limits), illustrative of the endocytic internalization of Mrp2 and Bsep induced by E_2_17G (2 µmol/liver), and its protection by the ERK1/2 inhibitor PD98059 (PD; 5 µM) or the p38 inhibitor SB203580 (SB; 250 nM). The lower panels show a densitometric analysis of the intensity of fluorescence associated with Bsep and Mrp2 along a 6 µm line perpendicular to the canaliculus (from −3 µm to +3 µm from the canalicular center), corresponding to the confocal images in the upper panels.

Prevention of the biliary secretory failure induced by E_2_17G by SB203580 and PD98059 was accompanied by the recovery of the normal canalicular localization of Bsep and Mrp2 at the end of the perfusion period (Fig. 6). In control livers, transporter-associated fluorescence was confined to the canalicular space (from −1 µm to +1 µm). In E_2_17G-treated livers, relocalization of intracellular fluorescence associated with both carriers from the canalicular space to the pericanalicular area was apparent, as indicated by the decrease in the fluorescence intensity in the canalicular area together with the increased fluorescence at a greater distance from the canaliculus (P<0.001 *vs.* controls); this indicates endocytic internalization of the carriers. Whereas SB203580 and PD98059 itself did not induce any changes in transporter localization (data not shown), both inhibitors extensively prevented the internalization of Bsep and Mrp2, as illustrated by a control-like pattern of the Bsep and Mrp2 distribution profiles in livers pretreated with the inhibitors, as compared with the E_2_17G-treated group (P<0.005 *vs.* E_2_17G; *n* = 20–50 canaliculi per experimental group, from three independent experiments). This supports our contention that p38 contributes to E_2_17G-induced cholestasis by retrieving canalicular carriers from their membrane domain in a cPKC-dependent manner. On the other hand, ERK1/2 would hinder the spontaneous retargeting of the endocytosed transporters by acting downstream PI3K.

## Discussion

This study provides further insights into the intracellular signalling pathways involved in E_2_17G-induced cholestasis.

First, we demonstrated here for the first time that E_2_17G activates the main MAPK types, *i.e.*, p38, ERK1/2 and JNK1/2 (see Fig. 1). Furthermore, the activation of p38 and ERK1/2 depended differentially on cPKC and PI3K, respectively. This differential dependency is in line with evidence in the literature showing a similar sequential activation, *i.e.* PI3K-ERK1/2 [12], [37], [38], [39] and cPKC-p38 [40], [41] in hepatocytes or HepG2 hepatoma cells.

**Figure 7:**
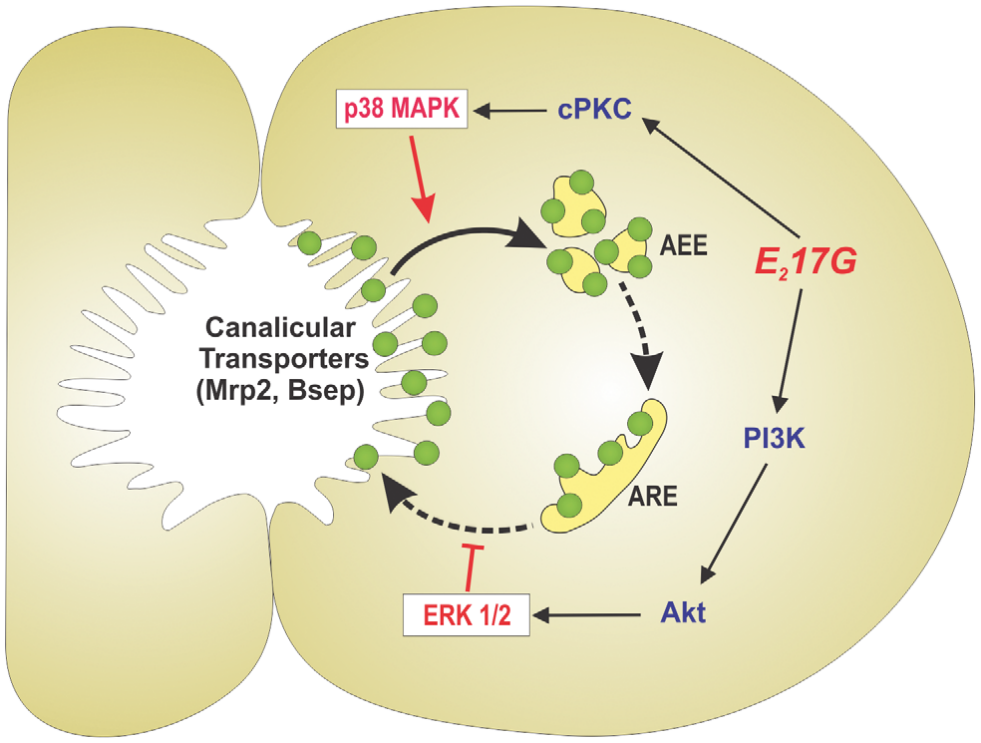
Schematic representation of the signalling events involved in E_2_17G-induced cholestasis by endocytic internalization and further retention of canalicular transporters relevant to bile formation (Bsep, Mrp2). p38, acting downstream of cPKC, triggers endocytic internalization of the apical carriers presumably towards apical early endosomes (AEE), the first intracellular endosomal compartment receiving internalized proteins from the apical membrane, in a microtubule-independent manner (solid arrow). These transporters traffic to, and accumulate into, apical recycling endosomes (ARE), from where they can be retargeted to the apical membrane during the recovery of the cholestatic process, in a microtubule-dependent manner (dashed arrows). Activation of the PI3K/Akt/ERK1/2 signalling pathway halts this latter process, thus explaining the increased colocalization of Bsep/Mrp2 with Rab11a, an ARE marker. This prolongs the cholestatic effect of E_2_17G by impeding the fast, spontaneous retargeting of intracellular transporters that would lead to a rapid recovery from the cholestatic injury.

The activation of these two signalling pathways by E_2_17G accounted for two independent and complementary cholestatic mechanisms; whereas the cPKC-p38 signalling pathway was essential for the acute cholestatic effect induced by E_2_17G, a phenomenon largely attributed to the endocytic internalization of Mrp2/Bsep, PI3K-ERK1/2 promoted the intracellular retention of these transporters during the cholestatic phenomenon (see Fig. 7). A similar complementarity had been demonstrated by us for their respective upstream activators, *i.e.* cPKC and PI3K/Akt [9].

The mechanism by which p38 mediates endocytosis results elusive because of our poor knowledge on the molecular mechanisms that trigger this process, but several reports in other cell models provide some hints. p38 accelerates clathrin-mediated endocytosis of several ligand receptors, such as epidermal growth factor receptor (EGFR) [42], the opioid receptors µ and δ [43], and the glutamate receptor [44], and there are several similarities between the internalization process of these receptors and that of Bsep. Like them, Bsep is internalized in a clathrin-dependent manner through a mechanism requiring the adaptor protein AP-2 [45], and AP-2 phosphorylation by p38 is essential for cargo recruitment to clathrin-coated pits, as shown for EGFR endocytosis [46]. Another putative p38 target is the Rab GDP dissociation inhibitor Rab:GDI. This protein stimulates the membranes/cytosol recycling of Rab5, a protein that coordinates formation of clathrin-coated vesicles and their fusion with early endosomes, thus increasing endocytic rate [47]. p38 increases Rab:GDI activity by direct phosphorylation at serine-121 [48]. Whether similar regulations for the endocytic process apply to Bsep, and whether Mrp2 shares with Bsep clathrin-mediated endocytic mechanisms remains to be ascertained. The latter is however probable, since endocytosis of Mrp2 in E_2_17G-induced cholestasis is a microtubule-independent event [7], and the same applies for clathrin-dependent endocytosis [49].

Our results also show that the PI3K/ERK1/2 pathway is involved in the intracellular retention of the endocytosed transporters, which otherwise would rapidly return to the canalicular membrane in a microtubule-dependent manner [7]. The nature of this vesicular trafficking is speculative at this point, but presumably involves the latest, microtubule-dependent exocytic step of the transcytotic route, which occurs via apical recycling endosomes (ARE). This is supported by the facts that: *1*) Bsep and Mrp2 colocalize with the ARE marker Rab11a (see Fig. 4), *2*) Mrp2 and Bsep colocalize on the same vesicles with both the microtubule motor dynein and polymeric immunoglobulin receptor (pIgR), a surface protein that is transcytosed via ARE from the basolateral to the apical membrane [50], *3*) insertion of Bsep back to the canalicular membrane from ARE is a microtubule-dependent process [51], and *4*) ARE is interconnected in a microtubule-dependent manner with apical early endosomes (AEE), the first station on the endocytic pathway involved in apical endocytosis [49]. Due to the microtubule-dependent nature of the exocytic process, microtubule-based motor proteins are a likely target for ERK1/2 modulation of canalicular transporter exocytosis. In line with this, ERK1/2 impairs binding activity of the microtubule motor kinesin-1 [52]; this protein is enriched in both AEE and ARE in liver (40% and 45% of total kinesin content, respectively) [53], and may therefore play a key role in the trafficking of canalicular transporters from AEE to ARE and in their exocytosis from ARE, which are both microtubule-dependent events [49].

The dependency of E_2_17G-induced cholestasis on both p38 and ERK1/2 results somewhat paradoxical with respect to other reports showing that both MAPK types are involved in choleretic phenomena, such as those induced by the bile salt tauroursodeoxycholate (TUDC) [54], [55], [56] and cAMP [57]. For example, TUDCA stimulates biliary excretion of the choleretic bile salt taurocholate via an integrin-dependent dual signalling pathway that involves both Ras/Raf/MEK/ERK1/2 and Src/p38 signalling pathways [54], [55], [56], [58]; of note, activation of ERK1/2 by TUDC depends on PI3K [59] but not on PKC [55], in agreement with our results (see Fig. 1). The p38-mediated stimulation of taurocholate excretion by TUDCA was due to an enhanced Bsep trafficking from the Golgi compartment to the canalicular membrane [56], and the further insertion of Bsep into the apical membrane [54], in a microtubule-dependent manner. The reason for the paradoxical pro-cholestatic and pro-choleretic effects of MAPKs is unclear at present, but several possibilities arise. MAPK activation is a highly compartmentalized process, which requires activation and localization in the organelle of scaffold proteins to allow for the regional recruitment of MAPK cascade components [60]. In quiescent hepatocytes, Raf-1 and MEK (MAPKK of ERK) are restricted to early endocytic compartments [61]. Therefore, rapid effects on endosomal trafficking of a MAPK modulating agent like E_2_17G may be limited to this early endosomal pathway, whereas the influence on Golgi/post-Golgi pathway would require further localization of specific MAPK scaffold proteins at this site [62]. In addition, E_2_17G and TUDC may activate different signalling pathways apart from MAPKs that, by operating in concert with MAPKs, result in opposite final effects. For example, TUDC-induced acceleration of the Golgi/post-Golgi exocytic pathway depends upon dual activation of p38 and novel PKC isoforms [56], and we have shown that E_2_17G does not activate the novel PKC isoform ε [8]. Alternatively, in a cholestatic context, TUDC-induced MAPK-dependent choleretic mechanisms may not be operative. Actually, MAPKs do not mediate TUDCA anticholestatic effects in TLC-induced cholestasis [23]. Finally, mediation of choleretic and cholestatic effects by p38 may reflect a differential capability of cholestatic and choleretic compounds to evocate p38 isoforms with opposite effects. Only α and ß p38 isoforms are present in liver, and they have been shown to exert opposite effects in other cell types [63]. Importantly, stimulation of Mrp2 apical translocation by cAMP, which prevents E_2_17G-induced internalization of canalicular carriers [6], involves exclusively p38α [57]. It would be of interest to find out whether p38α activity is decreased by E_2_17G, and whether cAMP reverses this decrease as part of its anticholestatic action.

While further studies will be required to assess the specific molecular mechanisms mediated by ERK1/2 and p38 to impair the localization status of canalicular transporters in estrogen-induced cholestasis, our results strengthen the idea that there is a clear interplay between signalling cascades and intracellular trafficking in this cholestasis, and that both MAPKs are key players in its ethiology. Drugs that inhibit selectively MAPKs have been used in preclinical and clinical studies with reasonable success [64]. As far as our results correlate with clinical cholestatic situations in humans, they should help to envisage new, feasible therapeutic strategies to treat them.
